# Precise Transmission for COVID-19 Information: Based on China’s Experience

**DOI:** 10.3390/ijerph18063015

**Published:** 2021-03-15

**Authors:** Wenjie Chen, Wenbing Zhang, Lu Li

**Affiliations:** 1 Institute of Creativity and Innovation, Xiamen University, Zhangzhou Campus of Xiamen University, Longhai, Zhangzhou 363105, Fujian, China; chenwenjie@xmu.edu.cn; 2 Department of Arts & Design, Xiamen University Tan Kah Kee College, Zhangzhou Campus of Xiamen University, Longhai, Zhangzhou 363105, Fujian, China; 3 Art College, Xiamen University, Siming Campus of Xiamen University, Siming, Xiamen 361005, Fujian, China; LL814472622@126.com

**Keywords:** data visualization, COVID-19 pandemic, pandemic map

## Abstract

Information on coronavirus disease 2019 (COVID-19) has been a significant focus for the global public since the outbreak of the disease. In response, data visualization has become the main form of media used to inform the public about the global pandemic’s progress. This paper studies the example of China, the main country affected by the virus in the early stage of the pandemic, to explain the problems regarding the differences in time, knowledge, and technology for information transmission. This paper also tries to explain the causes behind the dissemination of rumors, misjudgment of the public, and the difficulties of perception regarding pandemic information based on the three aspects of information collection, processing, and presentation. We argue that comprehensive information transmission with direct and clear visual presentation could help the public better understand the development of the pandemic, relieve social panic, and help authorities promptly adjust public health policies to absorb the social and economic impacts of the pandemic. Based on a case study, we propose that hierarchical presentation, comprehensive descriptions, and accurate visualizations of pandemic data can effectively improve information transmission, thus providing helpful references for authorities and organizations to improve the effectiveness of pandemic information transmission.

## 1. Introduction

At the end of 2019, coronavirus disease 2019 (COVID-19) became a global public healthcare emergency. China’s response to the pandemic was one of the quickest [1,2]. From 23 January 2020, when a lockdown in Wuhan, China, was declared, until 18 March 2020, when the numbers of new confirmed cases, new suspected cases, and current suspected cases were all reduced to zero, China experienced a full and difficult cycle of the pandemic. Numerous scholars have conducted substantial studies to model the spread of the virus and the effects of public healthcare interventions [3,4,5,6,7,8,9,10,11]. However, very few of these studies have paid attention to pandemic data visualization as a type of media to inform the public about the development of the pandemic. Data visualization is a form of visual art that uses graphics and shapes to represent quantified information and thus help the public understand the data. Although many efforts in data visualization have been made by the media and authorities to make data clear and understandable [12,13,14,15,16,17], accurate information transmission through data visualization has not been a widespread concern [18].

Among many types of pandemic data visualization are possible, the pandemic map, due to its geographic spatial attributes, has become the main type of media used by major organizations to notify the public of the pandemic situation and its development [19]. In this study, we used data published by the National Health Commission of the People’s Republic of China (NHC) from 11 January 2020 to 1 May 2020 to assess the pandemic maps released by the three major pandemic data release platforms: DXY.cn, AliHealth, and Baidu. The study revealed that there were three main problems for pandemic information transmission in China: the information lag and asymmetry caused by differences in the time and space of data disclosure can provide space for the breeding of rumors; the information disclosure methods adopted by data release platforms primarily employing absolute values, such as the number of confirmed diagnoses, can cause cognitive biases among the public and spread social panic; and the improper graphical representation of data visualization makes it difficult for the public to read and accurately understand the relevant information.

A complete model for information transmission includes information collection, processing, presentation, and reception. The accurate transmission of information requires the simultaneous integration of information collection, information processing, and information presentation. Only on the basis of timely, comprehensive, and accurate information organization and processing can the accurate transmission of information to recipients be achieved. Taking the pandemic map as an example, this paper illustrates the problems in the transmission of pandemic information in China under the analytical framework of the accurate transmission of pandemic information. We show that in the process of information collection, promptness should not only be reflected in the rapid disclosure of pandemic data by information providers but also in the rapid presentation of detailed geographic information of confirmed cases in a given area. When processing information, “comprehensive” means that in the provision of pandemic information, information processors should consider the size of the urban population, especially the city’s medical resources and capabilities, alongside other important factors. The “accuracy” of information presentation means that the way the data are presented will affect the public’s ability to quickly access effective pandemic information; thus, the accuracy of information transmission in data visualization should be improved. This study found that the three strategies of the hierarchical presentation of data, the comprehensive description of information, and the accurate expression of visualization can improve the status of information collection, information processing, and information presentation in the information transmission model. Not only can these factors improve the transparency of pandemic information, correct the audience’s misunderstanding, and enhance the public’s awareness of prevention, but they can also improve measures of pandemic prevention and control to make them more scientific, precise, and efficient for government. With the COVID-19 pandemic still raging around the world, this argument has important practical significance.

## 2. End of Rumors: Hierarchical Presentation for Pandemic Data

According to China’s Regulations on Preparedness for and Response to Emergent Public Health Hazards, emergency monitoring agencies, medical and health institutions, and relevant units should, when the outbreak of an infectious disease or epidemic occurs or is likely to occur, report to the local health authority within two hours; the health authority should also report to the local people’s government within two hours and, at the same time, report to the health authority of the higher people’s government and the health authority of the State Council. The Regulations on Emergency Responses to Public Health Emergencies clearly stipulate that pandemic information can only be released by the health authority of the State Council or local government health authorities with authorization from the health authority of the State Council. Since 11 January 2020, after the outbreak of COVID-19, the NHC, as the national authority, has released national pandemic data on a daily basis through its official website. The pandemic data provided by regions and provinces have been dynamically updated through three online platforms: DXY.cn (accessed on 22 February 2020), AliHealth, and Baidu. However, this method of disclosure has certain problems.

From the perspective of pandemic prevention and control, only disclosing pandemic information at the provincial (autonomous region, municipality) level has a limited effect on the vast majority of ordinary people with a limited living radius. However, the pandemic information at the district, county, and sub-district levels that can truly help the public take the necessary measures has never been promptly provided through official channels. The problem of the spatial gap that exists in the processes of information collection and release has not been fully explored, which has provided time and room for the spreading of rumors to a certain extent and caused substantial economic losses [20,21].

It is fair to say that China’s pandemic map with provinces as divisional units had positive significance for attracting public attention in the early stages of the pandemic [22]. However, as the pandemic developed, the cumulative number of confirmed cases quickly made the color of the provincial pandemic map turn “red” and “dark”. The public not only began to panic but also showed undifferentiated hostility towards vehicles and people from the same province in areas with varying levels of pandemic severity. Compared with the overreaction of the general public, local governments have adopted undifferentiated “one size fits all” or overlapping over-prevention and control measures, which have increased the government’s costs in fighting the pandemic and are not conducive to the central government’s need to coordinate pandemic prevention and control with social and economic development [23,24].

**Figure 1:**
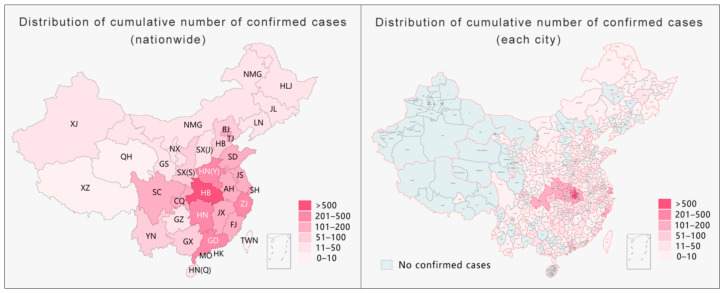
Comparison of the visualization results of the pandemic data at the provincial and municipal levels (as of 30 January 2020).

Taking the data released on 30 January 2020 by NHC as an example, the use of provincial-level pandemic data for visualization led to a situation where one diagnosis illuminated the entire map of the Tibet Autonomous Region [25]. However, by narrowing the administrative divisions for visualization to the level of cities, there was only one case in Lhasa city in the entire Tibet Autonomous Region; indeed, this case remains the only confirmed case in Tibet to date. As shown on the right side of Figure 1 below, by narrowing the administrative divisions for visualization, half of China could be exempted from severe prevention and control measures such as a “city lockdown”, “village lockdown”, or “road lockdown”. Moreover, if the scale were narrowed to the level of county and district, more areas could be exempted from excessive prevention and control measures. As the only confirmed case in Tibet was found in the Chengguan district of Lhasa city, the daily social and economic life in other parts of Tibet should not have been affected by pandemic prevention and control measures. Thus, during the pandemic, reducing the administrative division of the pandemic map for information disclosure would have not only greatly improved information transparency and effectively reduced the spread of rumors but would have also helped the government to implement differentiated prevention and control measures to maintain the normal production and life of society to the maximum level. The hierarchical presentation of pandemic data, as the key element in the process of information collection and release, is not only an effective way to fill gaps in pandemic information, improve data transparency, and dispel rumors but is also an important prerequisite for the implementation of differentiated-but-scientific prevention and control using a proper strategy. Without hierarchical information to provide a source of confidence, the government’s efforts to restore social life and resume production as soon as possible will be negatively affected [26].

## 3. Rational Judgment: A Comprehensive Description of the Pandemic Data

On 20 January 2020, the NHC included COVID-19 in the second level of notifiable diseases and adopted prevention and control measures for COVID-19 as a first level disease. In general, the pandemic data released by the NHC usually include three sections: (1) the new data (domestic/on-arrival newly confirmed diagnoses, new deaths, newly suspected diagnoses, newly cured diagnoses, and new asymptomatic diagnoses); (2) the current data (current domestic confirmed diagnoses, current on-arrival confirmed diagnoses, current suspected diagnoses, and current close contacts under medical observation); and (3) the cumulative data (cumulative confirmed diagnoses, cumulative cured diagnoses, cumulative death cases, and cumulative close contacts). The public can only rely on the above-mentioned limited data to infer the local pandemic situation, making it difficult to obtain an objective understanding of the true severity of the pandemic. The data themselves are “objective”, but the presentation of those data is the result of a human process of “judgment-identification-selection” [27]. Therefore, the processing of information has an important influence on the public’s comprehensive understanding of the severity of the pandemic.

The evolution of the pandemic in many countries shows that relevant trends are closely linked to the region’s population structure (especially age structure), population density, level of medical resources, and social distance [28,29,30,31]. The proportion of the number of confirmed cases to the total population is an important indicator to measure the severity of the pandemic in one region. The current level of healthcare resources is one of the key factors affecting the mortality rate. The number of hospitals (designated hospitals for COVID-19 treatment and other hospitals), the number of medical personnel (fever outpatient departments, emergency departments, infectious disease departments, respiratory medicine departments, and other emergency deployments), and the number of hospital beds (isolation wards, intensive care units, and observation rooms) are also key indicators for evaluating the level of the pandemic crisis in a region [32,33]. During the outbreak of the pandemic, the NHC organized 344 national medical teams, and 42,322 medical personnel in total were calculated up to 1 March 2020. To address the issue of the shortage of hospital beds, the local government built two temporary infectious disease hospitals and 20 field hospitals (Fangcang hospitals), providing over 30,000 hospital beds in a very short time. This measure effectively improved the doctor-to-patient ratio and was the main reason why the pandemic in Hubei was under control in a short time.

Our study argues that the relative number of cases obtained after comprehensively calculating the number of cases and related factors such as local population and local medical resources can describe the degree of the crisis in each region more objectively. Taking the pandemic data released by Jiangsu Province of China on 22 February 2020 as an example, after analyzing the results of both the relative and absolute numbers of confirmed cases, it was found that the two created different perceptions of the degree of the pandemic crisis among the public. (Absolute number confirmed diagnoses refers to the raw data of the confirmed diagnoses. The relative number of confirmed diagnoses was comprehensively calculated based on raw data and relative medical resource values in each region). This difference could mislead public health policy.

In the following section, the method adopted by this study is explained. First, key indicators such as the number of confirmed cases per capita, the number of hospitals per confirmed case, the number of doctors per confirmed case, and the number of hospital beds per confirmed case in each city of Jiangsu Province are calculated based on provincial statistics of the population of Jiangsu. Second, the relative number of confirmed diagnoses in each city of Jiangsu Province was calculated based on the values in Nanjing city as a reference. Finally, the above-mentioned confirmed numbers are added to obtain the pandemic crisis index of each city in Jiangsu Province on 22 February 2020. The larger the accumulated value is, the higher the degree of the pandemic crisis in the corresponding city (see Table 1 below).

Calculation formula: the relative number of confirmed diagnoses in city A = the proportion of city A/the proportion of Nanjing × the number of confirmed diagnoses in Nanjing. Taking the calculation of the relative confirmed number of cases based on the number of cases per capita in Suzhou as an example, the formula is x = 0.0814/0.1116 × 93, and x ≈ 68 cases are obtained. Then, the relative number of confirmed cases in each city can be calculated based on the proportion of the population of permanent residents, the number of hospitals, the number of doctors, and the number of hospital beds.

**Figure 2:**
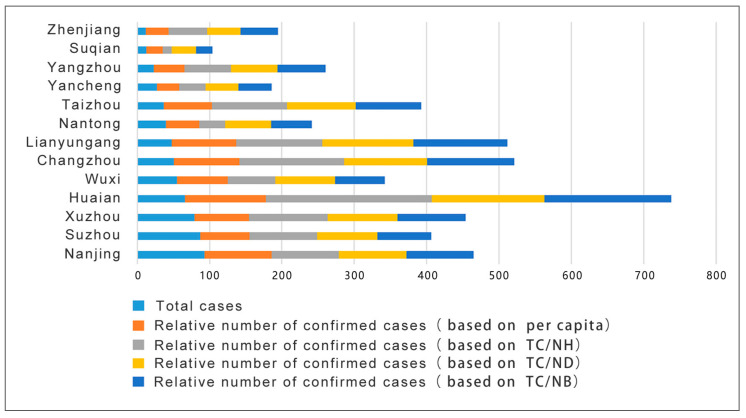
Pandemic crisis index map of each city in Jiangsu Province.

To better present such results, the data in the table were visualized to generate a pandemic crisis index map of each city in Jiangsu Province for that day (see Figure 2 below). It is clear that if the degree of the pandemic crisis in each city is measured according to the absolute number of diagnoses, where the crisis index of Nanjing is the highest. However, by measuring the relative number of confirmed cases, Huai’an is shown to have the highest index in terms of the number of cases per capita, the number of hospitals per capita, the number of doctors per capita, and the number of beds per capita. Although Nanjing accounted for the most daily confirmed cases, as the capital city of Jiangsu, its medical resources and capabilities are far better than those of a small city like Huai’an. This is the main reason for the differences in the results. The direct consequence of misjudging the pandemic crisis index in each city is the misallocation of already-scarce medical resources, which will negatively impact pandemic prevention and control.

**Figure 3:**
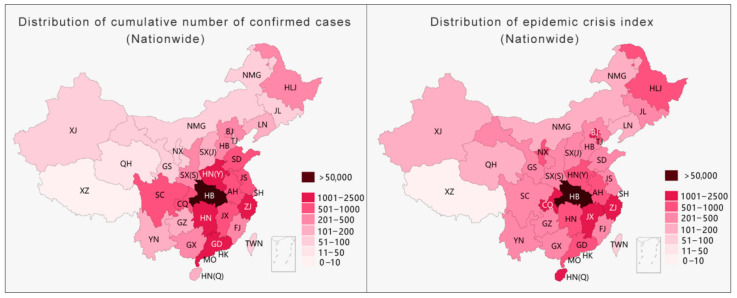
Comparison of the conclusions between the absolute and relative confirmed numbers (as of 22 February 2020). Note: The data on the left came from DXY.cn, accessed on 22 February 2020; the population data on the right are taken from the population statistics of 2019.

The same method was applied to calculate the pandemic crisis index for each province in China to determine if the results are generally applicable. The left side of Figure 3 illustrates a distribution map of the cumulative number of notified diagnoses nationwide on 22 February 2020. Hubei Province had the largest number of confirmed cases, followed by Henan Province, Hunan Province, Guangdong Province, and Zhejiang Province. On the right side is a crisis index graph calculated based on the number of cases per capita. (As medical data for most provinces in China are difficult to acquire, only the number of cases per capita for each province was used in this example.) Hubei Province still has the highest crisis index here, followed by Jiangxi Province, Chongqing Province, Hainan Province, and Zhejiang Province. (In this example, the value from Hubei was used as a reference to infer the number of cases per capita for the other provinces.) Similar to the results of the analysis of cities in Jiangsu Province, this study shows that the ranking of provinces based on different methods has significant differences.

While processing the collected raw pandemic data, Chinese health authorities simplified the comprehensive requirements for information release in the Regulations on Preparedness for and Response to Emergent Public Health Hazards. While the absolute number of confirmed diagnoses can roughly describe the pandemic infection situation of each administrative division unit, directly presenting key information such as the pandemic hot zones, the comprehensive multifactor relative number of confirmed diagnoses can conclusively describe and accurately evaluate the degree of the pandemic crisis in each region and can quickly recognize the provinces and cities at risk. For the government, this is helpful for creating long-term pandemic prevention and control policies and economic development plans by assessing the developing pandemic trends through the absolute number of diagnoses, while determining the pandemic crisis index through the relative number of diagnoses can avoid the misallocation of resources (especially scarce resources such as medical supplies). Given that the sudden outbreak of the pandemic caused a global shortage of medical supplies, a comprehensive description of pandemic information will help improve the pertinence and timeliness of government resource allocation. For the general public, understanding the degree of the pandemic crisis index in a city is meaningful for daily life.

## 4. Easy Reading: Accurate Expression of Pandemic Data

Information presentation is one of the most prominent problems in information transmission for China and many other countries. Data visualization is the main visual medium for pandemic information presentation, and pandemic maps are the main method for data visualization. Since data reporting and publications are based on the administrative divisions in China, pandemic maps characterized by hierarchical statistical maps and bubble maps have been widely used. The most commonly used type of hierarchical statistical map is a visual expression of the data variables mapped through color gradients. As the map embodies geographic division information, it has the advantage of effectively strengthening the public’s awareness of the relationship between the pandemic and geographic space. However, with the rapid development of the pandemic, the limitations of this method gradually emerged. There are three main limitations. First, because the color itself does not convey quantitative information, the reader cannot intuitively perceive the value of the mapped area through its color. Second, areas on the map filled with color lead the audience to perceive a greater density, rather than quantity, which is likely to cause cognitive bias [34]. Moreover, provinces at the same level but with larger areas are more likely to give readers the misconception that the pandemic situation is more serious than reality [35]. Third, during the outbreak of a pandemic, it is often necessary to continuously adjust the classification differences or increase the level of classification on the chart to reflect dynamic changes in the data, which is likely to cause fatigue in terms of discrimination and recognition ability.

**Figure 4:**
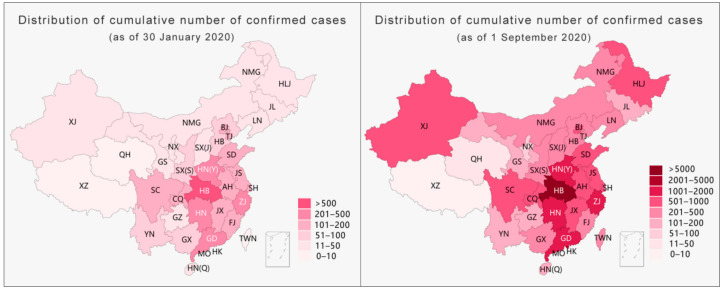
Method of increasing the level of classification.

The hierarchical statistical map of China’s pandemic situation usually adopts a color gradient from light to dark to map the severity of the pandemic from mild to severe. The color gradient only reflects the relative relationship between objects (this relative relationship is sometimes unclear) and cannot reflect quantities. Thus, such a map needs a separate reference for the numbers related to each color. In this way, except for the lightest and darkest colors, which are the most obvious, the different color levels in between are likely to cause confusion in the mapping relationship. Taking the left side of Figure 4 as an example, except for the darkest part, it is difficult to distinguish the differences between the other parts, and it becomes even more difficult to relate the colors to their corresponding numbers. With a rapid increase in the number of diagnoses, one way to present such data is by increasing the level of classifications. This method seems to provide more detailed pandemic information, but, in fact, readers’ recognition abilities decrease as the level of classification increases [36]. Taking Figure 4 as an example, with the same level of difference, after the classification level increases from the six levels on the left to the nine levels on the right, not only is the color difference between the areas reduced, but the effective association with the corresponding numbers is also greatly weakened. Increasing the number of grading methods appears to provide more information but actually diminishes what readers can realistically obtain.

**Figure 5:**
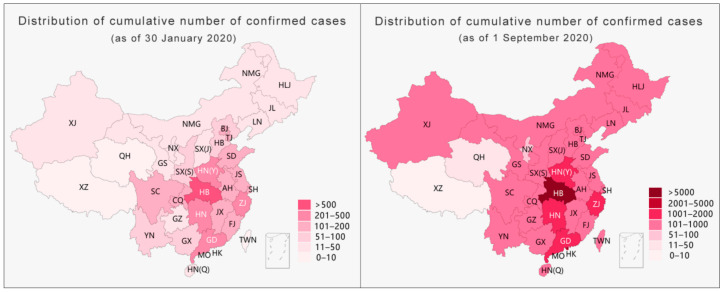
Method of increasing the level of difference.

With the level of classification remaining unchanged, the second method for dealing with extreme changing values is to increase the difference between each level (Figure 5) while maintaining the number of levels to ensure that the legibility of the visualization is applicable when the number is relatively small at the beginning of the pandemic. Nevertheless, as the current pandemic entered a period of rapid growth, the method of increasing the level of difference resulted in an inaccurate presentation of the differences in the pandemic data of each province, showing a trend of averaging the situation (right side of Figure 5).

**Figure 6:**
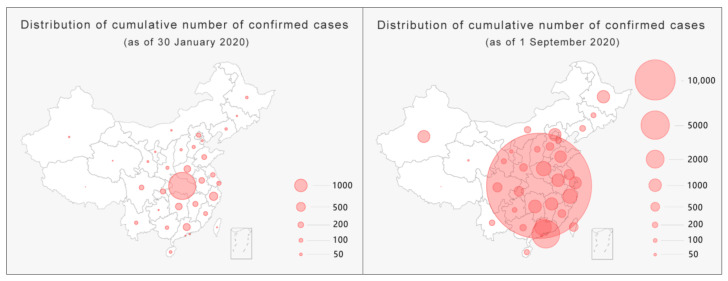
Visualization of a bubble map.

Another major form of pandemic maps is bubble maps (Figure 6), which map data variables through the size of the bubble area and act as a visual representation of a bubble chart combined with geospatial information. Through a comparison of the sizes of bubbles and tracking their changes, this type of map has the advantage of letting the audience intuitively perceive the development of the pandemic in different provinces and cities. However, it has two major shortcomings. First, when the value is too large, overlapping bubbles will weaken the correlation with the mapped area, causing visual confusion. Second, like hierarchical statistical maps, the relationship between bubbles and the corresponding numbers in the map is weakened by increasing the level of the classification or difference. Readers can only roughly infer the situation of the pandemic in geographic space by observing the relative gathering of bubbles.

In general, the explosive growth in the number of COVID-19 infections makes it difficult for both hierarchical statistical maps and bubble maps to accurately and effectively transmit pandemic information and even weakens the advantages of the geographic information attributes in pandemic maps. Specifically, the loss of the correlation between data and the mapped area and the inherent contradiction between data growth and precise expression are the two main challenges in pandemic data visualization and are also the problems that pandemic maps should focus on solving.

The present study divided classification in China into four levels: province (including autonomous regions and direct-administered municipalities), city, district/county, and street (village), corresponding to the four images numbered 1, 2, 3 and 4 in the figure below. For the provincial and municipal levels, we used bubble maps to visualize the development of the pandemic situation in each district. The borders of the original bubble map were removed while preserving the geographical and spatial relationships to avoid visual confusion caused by overlapping. At the district/county and street/village levels, detailed pandemic information is disclosed through hierarchical statistical maps. Given that the amount of data is small, the accuracy could be improved by reducing the levels of classification and decreasing the differences. Based on this visualization method, by using comprehensive information disclosure from large geographic spaces to small living spaces, not only can the spatial differences in information transmission be diminished, but the transmission of key information can also be more direct and accurate.

**Figure 7:**
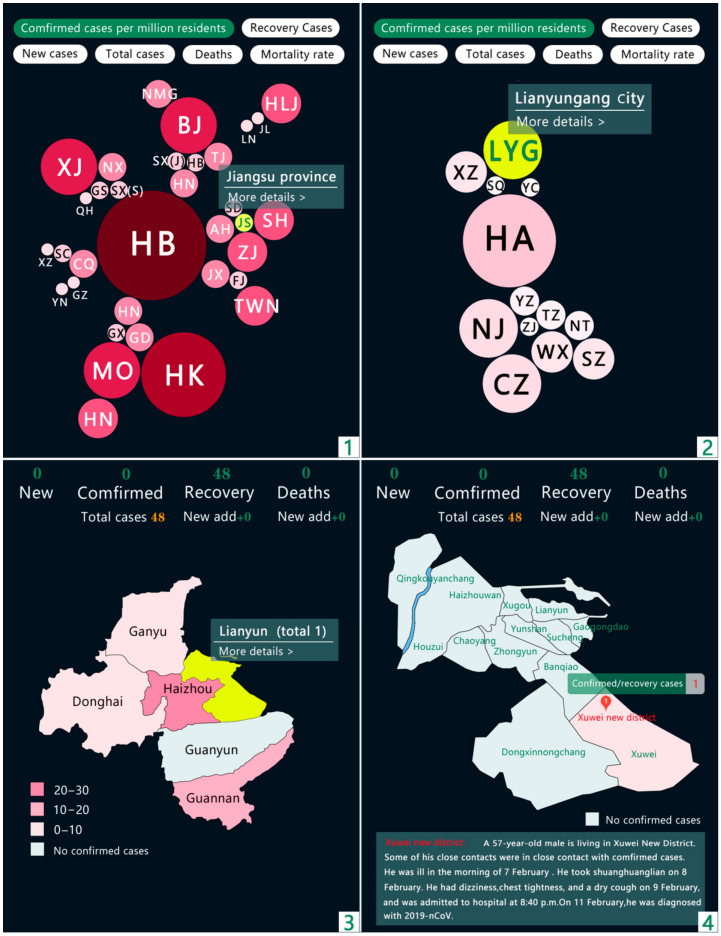
Content architecture of the pandemic situation’s visualization (as of 1 September 2020).

Regarding the disclosure of information about the pandemic situation (see Figure 7), the present study sought to change the single evaluation dimension based on absolute values, such as the number of confirmed diagnoses. Combining absolute values and relative data can describe and present the development of the pandemic more comprehensively and objectively. However, due to the difficulties in obtaining public health data in various places in China at this stage, we used the number of cases per million people as the basis for the classification levels of the pandemic and used several sets of bubbles of different sizes and colors to express the severity of the pandemic in cities and provinces. Each selected city highlights and provides further detailed information through interactions.

## 5. Discussion

As of 25 February 2021, the confirmed global cases for COVID-19 have reached over 110 million, with a death toll of over 2.5 million people. As a global health crisis, COVID-19 is a focus area for everyone on Earth, thus demanding higher quality pandemic information transmission. As one of the first countries to report on COVID-19, China has taken strict measures and active medical interventions for months and managed to turn the tide of the disease’s development in China with obvious positive effects. While other countries are still struggling with COVID-19, China has become one of the first countries to restore its normal social and economic order. However, China’s success in pandemic prevention and control cannot completely cover its shortcomings in the transmission of pandemic information. This problem is also common in countries like the United States, where the pandemic is the most severe, and has a certain universality.

The information collection stage in the early period of the pandemic; the uncertainty in understanding the core issues in China, such as the mechanisms of the new coronavirus, the scale of the infected population, and whether there is human-to-human transmission; and the lack of an ideal structure for pandemic information reporting mechanisms and information release platforms caused delays in the administrative decision-making process and information release. Such delays caused the public to have an exaggerated understanding of the risks and consequences of infection, leading to the birth of rumors and their rapid spread through social media [37]. Thus, in terms of legislative development, we believe that the Regulations on Preparedness for and Response to Emergent Public Health Hazards should not only provide strict regulations on the time limit for reporting epidemic information but also mandate the time limit for releasing epidemic information. Closing the time gap for public information access, from a legal perspective, is the practical purpose of implementing the Regulations on Preparedness for and Response to Emergent Public Health Hazards for timely information release. It was concluded based on observations that the time delays in disseminating information to the public could be eliminated if the NHC had a more efficient pandemic information reporting mechanism and if the information could be processed better. Furthermore, taking the lack of confidence of the public in some local authorities and media into account, if the NHC were to be more specific when releasing pandemic data at the city, county, and street levels, not just at the province level, then information transparency would be significantly improved, and the issue of pandemic data differences in space could be effectively addressed. It is clear from the reports of domestic diagnoses since October 2020 that authorities have realized the issue of pandemic data differences in space; however, no effective changes have yet been seen in the released pandemic maps. As an example, although substantial pandemic information is provided on the CDC’s website, in terms of specific location and space, the administrative information provided by the CDC only reaches the levels of counties, which is basically the same as the level of information provided by the Chinese NHC.

When collecting and reporting pandemic information, information release agencies should not only remain at the provincial level but should divide levels into cities, counties, and subdistricts and present key geographic pandemic information hierarchically. While significantly improving the transparency of pandemic information, the problem of spatial gaps in pandemic data should also be eliminated. This would allow data to truly reflect the requirements for accurate information release in the Regulations on Preparedness for and Response to Emergent Public Health Hazards. Compared to the time differences in pandemic information, the problem of spatial differences is a more common phenomenon in the transmission of pandemic information.

In the information processing stage before the summary and release of the pandemic data, the dimensions of China’s pandemic data were too simple. Raw data that do not consider key factors such as the medical level and population can easily distort the pandemic crisis index of a city and cause misunderstandings. The comprehensiveness requirements for information release in the Regulations on Preparedness for and Response to Emergent Public Health Hazards have not, therefore, been accurately reflected. Changes in the information environment can increase the effectiveness of non-pharmacological interventions [38]. Therefore, the population size and medical resources of cities in China are considered to be important influencing factors. This is an important element neglected by the Chinese public health authorities in the transmission of pandemic information, with the consequential risk of misallocating scarce medical resources (Figure 2). This risk is not unique to China but also exists in developed countries such as the United States, which have relatively complete pandemic data records. Although the US CDC far exceeds the NHC in terms of providing pandemic data [39], in terms of medical resources alone, the CDC, like the NHC, does not provide effective data and evaluation. However, this indicator is very important for evaluating the pandemic crisis index in a city.

Regarding pandemic information presentation, for both the hierarchical statistical maps and the bubble maps, it is clear that the health authorities in China remain deeply affected by the perspective of paper media mapping. Although substantial changes have been made to the forms and colors of pandemic maps, there is still the problem of mismatches between the image and the information. We studied the pandemic maps from the CDC. Although their color patterns are different to those of China and apply more interactive technologies, maps from the CDC still show problems in the relationship between the image and its related information. Thus, the present study proposes a method that integrates the advantages of both hierarchical statistical maps and bubble maps. By building interactive images for the four administrative division levels, the pandemic information in each division, from large to small scales, can be clearly presented. This would strengthen the transparency and feasibility of the pandemic information in each division.

It should be noted that the main subject of this study is not the most typical one at the moment. The present study only focused on building a theoretical structure for accurate information transmission and the visualization of pandemic maps. No further discussion was included to explore the technology behind these maps. Compared to other prevention and control measures that have proven effective, the effects of accurate information transmission are not apparent. In addition, the pandemic information transmission varies widely in different countries, and this difference may make our method partially ineffective. However, it is believed that analyzing and reviewing sections of the pandemic information transmission in China, as well as specific discussions on the content and standards of information release, will help governments and institutions establish effective information release mechanisms and better deal with the present public health crisis.

## 6. Conclusions

The Regulations on the Preparedness for and Response to Emergent Public Health Hazards from Chinese authorities outline the three general principles of providing “timely”, “accurate”, and “comprehensive” information but without detailed and operable standards. Moreover, it is difficult to effectively execute these guidelines in pandemic information transmission. After analyzing three sections of information transmission, this study identified and summarized the existing problems related to information transmission in China and proposed a specific method to meet the requirements of timely, accurate, and comprehensive information transmission. It was shown that the hierarchical presentation of pandemic information could correct the spatial differences in information transmission. Based on the pandemic crisis index calculated from the relative number of confirmed cases, the allocation efficiency for medical resources could be improved, and objective public awareness for pandemic development could be increased. Through accurate data visualization, excessive prevention measures could be avoided by correcting inaccurate perceptions among the public. From the perspective of public policy, the accurate transmission of pandemic information is a precondition for authorities to implement precise pandemic measures and also the foundation to direct the public to cooperate with such measures. Information transmission should focus more on individual needs for information. This would increase confidence in the restoration of social order when the development of the pandemic indicates a positive trajectory. For each individual and family, being updated on comprehensive pandemic information, remaining alert, staying safe, and maintaining social distance still have practical importance.

## Figures and Tables

**Table 1:** Statistics of absolute and relative confirmed diagnoses among the cities in Jiangsu Province on 22 February 2020.

City Name	TC	PRP (TT)	PC (PM)	RNCC	NH	$\frac{TC}{NH}$	RNCC	ND	$\frac{TC}{ND}$	RNCC	NB	$\frac{TC}{NB}$	RNCC
Nanjing	93	833.5	0.1116	93	222	0.4189	93	31,600	0.00294	93	49,448	0.00188	93
Suzhou	87	1068.36	0.0814	68	206	0.4223	94	32,900	0.00264	84	58,022	0.00150	74
Xuzhou	79	876.35	0.0901	75	161	0.4907	109	25,800	0.00306	97	41,553	0.00190	94
Huai’an	66	491.4	0.1343	112	64	1.0313	229	13,400	0.00493	156	18,597	0.00355	175
Wuxi	55	655.3	0.0839	70	185	0.2973	66	21,000	0.00262	83	39,743	0.00138	68
Changzhou	51	471.73	0.1081	90	78	0.6538	145	14,100	0.00362	114	20,927	0.00244	121
Lianyungang	48	451.84	0.1062	89	89	0.5393	120	12,100	0.00397	125	18,215	0.00264	130
Nantong	40	730.5	0.0548	46	246	0.1626	36	19,900	0.00201	64	35,425	0.00113	56
Taizhou	37	465.19	0.0795	66	79	0.4684	104	12,300	0.00301	95	20,253	0.00183	90
Yancheng	27	724.22	0.0373	31	164	0.1646	37	18,800	0.00144	45	29,155	0.00093	46
Yangzhou	23	450.82	0.0510	43	80	0.2875	64	11,200	0.00205	65	17,119	0.00134	66
Suqian	13	491.46	0.0265	22	229	0.0568	13	12,200	0.00107	34	28,108	0.00046	23
Zhenjiang	12	318.63	0.0377	31	50	0.2400	53	8200	0.00146	46	11,416	0.00105	52

TC: Total cases; PRP(T): Permanent resident population (per ten thousand); PC(TM): Per capita (per million); RNCC: Relative number of confirmed cases; NH: Number of hospitals; ND: Number of doctors; NB: Number of beds; The healthcare data in this table come from the 2018 Statistical Yearbook of Jiangsu Province; the number of confirmed cases comes from DXY.cn (accessed on 22 February 2020).

## Data Availability

The data presented in this study are openly available in website of National Health Commission of the People’s Republic of China.

## References

[1] National Health Commission of the People’s Republic of China, Use Full Efforts to Do Well the Job of Prevention and Control for COVID-19. http://www.nhc.gov.cn/xcs/fkdt/202001/d9570f3a52614113ae0093df51509684.shtml.

[2] Maier B.F., Brockmann D. (2020). Effective containment explains subexponential growth in recent confirmed COVID-19 cases in China. Science.

[3] Thomas D.M., Sturdivant R., Dhurandhar N.V., Debroy S., Clark N. (2020). A primer on COVID-19 mathematical models. Obesity.

[4] Li Q., Guan X., Wu P., Wang X., Zhou L., Tong Y., Ren R., Leung K.S.M., Lau E.H.Y., Wong J.Y. (2020). Early transmission dynamics in Wuhan, China, of novel coronavirus-infected pneumonia. N. Engl. J. Med..

[5] Wu J.T., Leung K., Leung G.M. (2020). Nowcasting and forecasting the potential domestic and international spread of the 2019-nCoV outbreak originating in Wuhan, China: A modelling study. Lancet.

[6] Hwang J., Park H., Kim S.-H., Jung J., Kim N. Basic and Effective Reproduction Numbers of COVID-19 Cases in South Korea Excluding Sincheonji Cases. https://www.medrxiv.org/content/10.1101/2020.03.19.20039347v2.

[7] Zhuang Z., Zhao S., Lin Q., Cao P., Lou Y., Yang L., Yang S., He D. (2020). Preliminary estimating the reproduction number of the coronavirus disease (COVID-19) outbreak in Republic of Korea and Italy by 5 March 2020. Int. J. Infect. Dis..

[8] Zhao S., Lin Q., Ran J., Musa S.S., Yang G., Wang W., Lou Y., Gao D., Yang L., He D. (2020). Preliminary estimation of the basic reproduction number of novel coronavirus (2019-nCoV) in China, from 2019 to 2020: A data-driven analysis in the early phase of the outbreak. Int. J. Infect. Dis..

[9] Law K.B., Peariasamy K.M., Gill B.S., Singh S., Sundram B.M., Rajendran K., Dass S.C., Lee Y.L., Goh P.P., Ibrahim H. (2020). Tracking the early depleting transmission dynamics of COVID-19 with a time-varying SIR model. Sci. Rep..

[10] Salim N., Chan W.H., Mansor S., Bazin N.E.N., Amaran S., Faudzi A.A.M., Zainal A., Huspi S.H., Hooi E.K.J., Shithil S.M. COVID-19 Epidemic in Malaysia: Impact of Lock-Down on Infection Dynamics. https://www.medrxiv.org/content/10.1101/2020.04.08.20057463v1.

[11] Cheng Q., Liu Z., Cheng G., Huang J. (2020). Heterogeneity and effectiveness analysis of COVID-19 prevention and control in major cities in China through time-varying reproduction number estimation. Sci. Rep..

[12] Gao P., Zhang H., Wu Z., Wang J. (2020). Visualising the expansion and spread of Coronavirus disease 2019 by cartograms. Environ. Plan. A Econ. Space.

[13] Zhang H., Chen Y., Gao P., Wu Z. (2020). Mapping the changing Internet attention to the spread of Coronavirus disease 2019 in China. Environ. Plan. A Econ. Space.

[14] Hong L. (2020). Visual design path of epidemic data from the perspective of the public. Pack. Eng..

[15] Loehrke J., Zarracina J., Petras G. Visualizing the Spread of the Coronavirus. USA Today.

[16] Mamoon N., Rasskin G. COVID-19 Visualizer. https://www.covidvisualizer.com/.

[17] Tableau. https://www.tableau.com/covid-19-Coronavirus-data-resources.

[18] Tuo L. (2020). Information visualization: A design method of making the Epidemic “visible” to the public. Zhuangshi.

[19] Yuanbo S., Zhiyi W., Ruige X., Fei L., Ge L. (2020). Data visualization design of COVID-19 epidemic. Pack. Eng..

[20] Koch C., Okamura K. Benford’s Law and COVID-19 Reporting.

[21] Jianzhang L. Don’t Lest Excessive Pandemic Prevention Measures Destroy China’s Economy. Caixin Weekly.

[22] Say No to “One Size Fits All” Pandemic Prevention and Control Measures. Overreaction of Prevention and Control Measures Must Be Corrected in Time. People’s Daily.

[23] Pan A., Liu L., Wang C., Guo H., Hao X., Wang Q., Huang J., He N., Yu H., Lin X. (2020). Association of Public Health Interventions with the Epidemiology of the COVID-19 Outbreak in Wuhan, China. JAMA.

[24] Chengying H., Yuechun W., Yali C., Xiaoxu G. (2020). Measurement and analysis of the COVID-19 epidemic inpact on China’s economy. J. Quant. Tech. Econ..

[25] National Health Commission of the People’s Republic of China. http://www.nhc.gov.cn/xcs/yqtb/202001/e71bd2e7a0824ca69f87bbf1bef2a3c9.shtml.

[26] Many Local Officials Take the Lead to Eat in Restaurants to Encourage Residents to Go Out and Consume. The Beijing News.

[27] Ambrosio C., Galavotti M., Dieks D., Gonzalez W., Hartmann S., Uebel T., Weber M. (2014). Objectivity and Visual Practices in Science and Art. New Directions in the Philosophy of Science. The Philosophy of Science in a European Perspective.

[28] Xian Y.F. Countries Fighting the Pandemic from the Perspective of Population and Aging. Global Times.

[29] Tingwei Z. (2020). Impacts of the built environment on the COVID-19 epidemic and the evidence-based practice: A preliminary analysis of the COVID-19 epidemic in American cities. City Plan. Rev..

[30] Shifu W., Cheng W., Yuan Y., Zhuoran S., Ke X., Jianzhong H., Tianyao Z. (2020). Academic discussions on human settlement environmental plan and design in the background of COVID-19 epidemics. South Archit..

[31] Moosa I.A. (2020). The effectiveness of social distancing in containing Covid-19. Appl. Econ..

[32] Jing L., Xiaoying L., Ling Z., Yongmei Z., Li X. (2020). Hospital nursing management system for emergency response to novel Coronavirus pneumonia outbreak. Nurs. J. Chin. People’s Lib. Army.

[33] Jinghui F., Zhongxiang C., Xin Y., Xuefen W., Yunyan X., Chunying L. (2020). Discussion on management model of nursing human resourses in response to new Coronavirus pneumonia. Chin. J. Respir. Crit. Care Med..

[34] Monmonier M. (2012). How to Lie with Maps.

[35] Fenglin S. (2020). Data visualization design for real-time report of epidemic map. Youth J..

[36] Yuexin Y., Xiujun L. (2018). Discussion on the hierarchical design method of statisticcal data in the statistical map of thematic map-taking “Hebei Provincial Atlas” as an example. Mod. Inf. Technol..

[37] Swire-Thompson B., Lazer D. (2020). Public health and online misinformation: Challenges and recommendations. Annu. Rev. Public Health.

[38] Charoenwong B., Kwan A., Pursiainen V. (2020). Social connections with COVID-19-affected areas increase compliance with mobility restrictions. Sci. Adv..

[39] CDC. https://covid.cdc.gov/covid-data-tracker/#cases_casesper100klast7days.

